# Serum anti‐GM2 and anti‐GalNAc‐GD1a ganglioside IgG antibodies are biomarkers for immune‐mediated polyneuropathies in cats

**DOI:** 10.1111/jns.12529

**Published:** 2023-01-16

**Authors:** Susan K. Halstead, Mark Jackson, Ezio Bianchi, Stefan Rupp, Nicolas Granger, Marika Menchetti, Greta Galli, Paul Freeman, Adriana Kaczmarska, Sofie F. M. Bhatti, Josep Brocal, Roberto José‐López, Andrea Tipold, Rodrigo Gutierrez Quintana, Edward J. Ives, Theofanis Liatis, Jasmin Nessler, Clare Rusbridge, Hugh J. Willison, Angie Rupp

**Affiliations:** ^1^ Neuroimmunology Laboratories, School of Infection and Immunity University of Glasgow Glasgow UK; ^2^ School of Cancer Sciences University of Glasgow Bearsden UK; ^3^ Department of Veterinary Science University of Parma Parma Italy; ^4^ Tierklinik Hofheim Germany; ^5^ Queen Mother Hospital for Animals, Royal Veterinary College University of London Hatfield UK; ^6^ CVS Referrals Bristol Veterinary Specialists Bristol UK; ^7^ Neurology and Neurosurgery Division San Marco Veterinary Clinic and Laboratory Veggiano Italy; ^8^ Queens Veterinary School Hospital, Dept of Veterinary Medicine University of Cambridge Cambridge UK; ^9^ Small Animal Hospital, School of Biodiversity, One Health and Veterinary Medicine University of Glasgow Glasgow UK; ^10^ Small Animal Department, Faculty of Veterinary Medicine Ghent University Merelbeke Belgium; ^11^ Anderson Moores Veterinary Specialists Hursley UK; ^12^ Hamilton Specialist Referrals—IVC Evidensia High Wycombe UK; ^13^ Department of Small Animal Medicine and Surgery University of Veterinary Medicine Foundation Hannover Germany; ^14^ School of Veterinary Medicine The University of Surrey Guildford UK; ^15^ Division of Pathology, Public Health and Disease Investigation, School of Biodiversity, One Health and Veterinary Medicine University of Glasgow Glasgow UK

**Keywords:** (max 5): feline, auto‐antibody, glycolipid, GM2, poly(radiculo)neuropathy

## Abstract

Recent work identified anti‐GM2 and anti‐GalNAc‐GD1a IgG ganglioside antibodies as biomarkers in dogs clinically diagnosed with acute canine polyradiculoneuritis, in turn considered a canine equivalent of Guillain‐Barré syndrome. This study aims to investigate the serum prevalence of similar antibodies in cats clinically diagnosed with immune‐mediated polyneuropathies. The sera from 41 cats clinically diagnosed with immune‐mediated polyneuropathies (IPN), 9 cats with other neurological or neuromuscular disorders (ONM) and 46 neurologically normal cats (CTRL) were examined for the presence of IgG antibodies against glycolipids GM1, GM2, GD1a, GD1b, GalNAc‐GD1a, GA1, SGPG, LM1, galactocerebroside and sulphatide. A total of 29/41 IPN‐cats had either anti‐GM2 or anti‐GalNAc‐GD1a IgG antibodies, with 24/29 cats having both. Direct comparison of anti‐GM2 (sensitivity: 70.7%; specificity: 78.2%) and anti‐GalNAc‐GD1a (sensitivity: 70.7%; specificity: 70.9%) antibodies narrowly showed anti‐GM2 IgG antibodies to be the better marker for identifying IPN‐cats when compared to the combined ONM and CTRL groups (*P* = .049). Anti‐GA1 and/or anti‐sulphatide IgG antibodies were ubiquitously present across all sample groups, whereas antibodies against GM1, GD1a, GD1b, SGPG, LM1 and galactocerebroside were overall only rarely observed. Anti‐GM2 and anti‐GalNAc‐GD1a IgG antibodies may serve as serum biomarkers for immune‐mediated polyneuropathies in cats, as previously observed in dogs and humans.

## 1 INTRODUCTION

Peripheral polyneuropathies and neuromuscular disorders, which may represent close to 20% of the neurological disease burden in cats (*Felis catus*)^1^ can present with overlapping clinical features and a variable clinical course.^2^, ^3^ Potential aetiologies include degenerative, inflammatory (infectious or immune‐mediated), metabolic, toxic, vascular, neoplastic/paraneoplastic or traumatic disorders. The list of differential diagnoses is thus wide and approached through combining information from the signalment, clinical history and neurological examination, with serological and metabolic screens, electrophysiological investigations, and nerve and muscle biopsies.

Poly(radiculo)neuropathies in cats considered to be of immune‐mediated aetiology accounted for nearly 60% of feline nerve biopsy material submitted for histological examination in a previous study.^4^ These conditions may present clinically in acute or chronic patterns, may affect specific anatomical regions, such as bilateral brachial plexus neuritis, and may be more prevalent in certain breeds, such as Bengal Cat Polyneuropathy.^2^ Bengal Cat Polyneuropathy typically affects young cats, may manifest with multiple episodes and a relapsing/remitting course, and a full or partial recovery is achieved in around 90% of cases. Electrophysiological and histological features comprise a demyelinating and distal denervating phenotype.^5^

Over recent years, a polyneuropathy with similar clinical and biopsy features to Bengal Cat Polyneuropathy, termed “Heterogenous Motor Polyneuropathy in Young Cats” has been observed in many other breeds, including Domestic Short‐ or Longhaired, Siamese and Persian cats.^6^ Birman and British Shorthair cats are also considered to potentially be predisposed to immune‐mediated polyradiculoneuropathies.^4^

In acute and chronic immune‐mediated peripheral neuropathies in man, including Guillain‐Barré syndrome (GBS), many anti‐glycolipid antibodies (AGAbs) have been identified as serological markers of disease.^7^, ^8^ Whilst anti‐GM2 and anti‐GalNAc‐GD1a antibodies are not the most common AGAb biomarkers in man, both are well‐described in acute and chronic auto‐immune neuropathy syndromes.^9^, ^10^, ^11^, ^12^, ^13^ Knowledge of human disease biomarkers in GBS led us to first investigate dogs clinically diagnosed with acute canine polyradiculoneuritis (ACP), a canine equivalent to GBS, for similar AGAbs. In ACP, initially in a pilot study and latterly in a larger international cohort, we observed a high prevalence of serum anti‐GM2 and anti‐GalNAc‐GD1a antibodies.^14^, ^15^ Herein, we investigated cats clinically diagnosed with immune‐mediated polyneuropathies (IPN) in comparison to neurological and non‐neurological control groups for the prevalence of similar serum AGAbs.

## 2 MATERIALS AND METHODS

### 2.1 Samples

Cat serum samples were acquired over 8 years (2015‐2022), following a national (UK) and international call for diseased and control serum samples sent out initially to board‐certified veterinary neurologists. The study was ethically approved by the University of Cambridge (CR101) and University of Glasgow (Ref14a/16).

The presumptive diagnosis of immune‐mediated polyneuropathy (IPN) was based on signalment (commonly young Bengal or other purebred cat, but also other cat breed, of any sex, with initial presentation typically at less than 1 year of age), and a clinical history of progressive (typically over 1‐2 weeks) or relapsing/remitting para‐ or tetraparesis, decreased or absent withdrawal reflexes in all limbs, and occasional hyperaesthesia and cranial nerve involvement (typical bilateral facial nerve paresis). The diagnosis was supported by ancillary investigations including an absence of biochemical abnormalities to explain the presenting clinical signs, negative infectious screens (eg, for *Toxoplasma gondii*, feline leukaemia virus (FeLV), feline immunodeficiency virus (FIV) and feline coronavirus (FCoV)), supportive electrophysiological changes, such as abnormal spontaneous myofibre activity on electromyography (EMG), decreased motor nerve conduction velocity and compound muscle action potential (CMAP) amplitudes, and variable conduction block,^5^, ^6^, ^16^ and the results of muscle/nerve biopsies when performed. Cases were excluded if there was a history of known toxin exposure or if a definitive aetiology other than IPN (such as metabolic deficiencies) was detected during workup to explain the presenting clinical signs.

Other neurological and neuromuscular (ONM) control cats comprised cats with central nervous system signs (such as seizures) and peripheral or cranial neuropathies and neuromuscular disorders other than IPN (such as myasthenia gravis). Neurologically normal control (CTRL) cats comprised cats undergoing investigations for diseases affecting other organ systems and lacking any evidence of neurological involvement.

All contributors were provided with sample submission guidelines, owner information sheets, consent forms and a questionnaire addressing patient demographics and epidemiological data (signalment [age, sex, and breed], date of disease onset, presentation and sampling), clinical features, preceding events (within 3 weeks of disease onset) and ancillary investigations carried out.

All samples were shipped with cooling agents, were immediately blinded by coding upon receipt and stored at −80°C until use.

### 2.2 Sample screening

All cat sera were screened in triplicate on combinatorial glycolipid array against a panel of 10 distinct glycolipids (GM1, GM2, GD1a, GD1b, GalNAc‐GD1a, GA1, SGPG, LM1, galactocerebroside and sulphatide) and their 1:1 (v:v) heteromeric complexes, each at 200 μg/mL. Each array contained duplicates of each unique antigen target in addition to solvent only (methanol) spots printed onto low fluorescence PVDF‐coated slides. Non‐specific binding sites were quenched by blocking slides in 2% bovine serum albumin (BSA) in phosphate buffered saline (PBS) for 1 hour at room temperature with gentle mixing, followed by application of a single dilute cat serum (1:50 in 1% BSA in PBS) per array, for 1 hour at 4°C. Anti‐glycolipid IgG was detected using Alexafluor 647 conjugated Goat anti‐Cat IgG (Fc specific) antibody (3 μg/mL; Jackson ImmunoResearch Laboratories, Pennsylvania). The median fluorescent signal associated with each unique target per serum was quantitated (Genepix 4300A microarray scanner, Molecular Devices, San Jose) including the subtraction of local fluorescence background signal and summarised as the mean of duplicate targets and expressed as fluorescence intensity unit (FIU).

Additionally, regardless of any previous investigations, all IPN samples were screened for an infection with FeLV, FIV, FCoV and *Toxoplasma gondii* by Veterinary Diagnostic Services, University of Glasgow, Scotland.

### 2.3 Data analysis and statistics

FIU values were converted to binary data with the application of an optimal cut‐off threshold calculated for each unique glycolipid target to discriminate between IPN and combined ONM and CTRL groups by plotting the ROC curve (MedCalc Statistical software version 19.7, Ostend, Belgium) and utilising the Youden index (J) method^17^ when giving equal weight to sensitivity and specificity. Both neurological/neuromuscular (ONM) and non‐neurological (CTRL) control groups were combined to allow for comparison of IPN to both neurological controls and to age−/breed‐specific control sera at the same time, bearing the distinct age‐ and breed‐specific picture (BCP) for many IPN‐cases in mind. The DeLong method^18^ was employed for the comparison of paired ROC curves. Array data for each cat serum was graphically displayed as heat maps using the rainbow scale to broadly distinguish intensity values across different glycolipid targets (MultiExperiment Viewer software; version 4.9.0; TM4 software suite).

Gaussian data were analysed using a two‐tailed Student's *t* test. Proportion data were analysed using Fisher's exact test for counts. Predictive associations between demographical, epidemiological and clinical factors, and antibody‐positivity (following thresholding of antibody into positive/negative and excluding any factors not recorded in more than 25% of the IPN‐population) were assessed by fitting a binomial logit generalised linear model. Factors to be included were determined by measurement of the Akaike information criterion and model significance confirmed using likelihood ratio testing. Statistical analyses were conducted in R (version 4.1.2; https://www.R-project.org/).^19^

## 3 RESULTS

### 3.1 Samples submission, signalment and clinical presentation

In total, 96 feline serum samples were submitted (41 IPN, 9 ONM, 46 CTRL; Table 1) from five European countries (UK, Italy, Germany, Belgium and Switzerland). All 41 IPN‐samples were accompanied by clinical questionnaires, which indicated that in 34/41 IPN‐cats the clinical and neurological examinations had been supported by electrophysiologic investigations, in 23/41 IPN‐cats by infectious diseases screens, in 17/41 IPN‐cats by muscle/nerve biopsies and in 16/41 IPN‐cats by examination of the cerebrospinal fluid.

**TABLE 1:** Patient demographics of feline serum samples

	IPN	ONM	CTRL	p (IPN vs ONM + CTRL)
Total samples	41	9	46	
Male (%)	31 (75.6)	3 (33.3)	26 (56.5)	*P* = .0334
Female (%)	10 (24.4)	6 (66.7)	19 (41.3)	
Unknown	0	0	1 (2.2)	
Age mean (median; months)	21.1 (9)	21.4 (18)	18.9 (12)	*P* = .9070
Age range	4 mo‐9y4mo	4 mo‐8y	4 mo‐5y	
Cats up to 2y (including; %)	29 (70.7)	8 (88.9)	34 (73.9)	*P* = .6396
Most common breeds (%)				*P* = .0038
Bengal	12 (29.3)	0 (0)	6 (13.0)	
Domestic Shorthair	11 (26.8)	6 (66.7)	29 (63.0)	
British Shorthair	8 (19.5)	0 (0)	8 (17.4)	
Persian	3 (7.3)	1 (11.1)	0 (0)	
Maine Coon	2 (4.9)	0 (0)	2 (4.3)	

*Note*: Other breeds: Abyssinian (n = 1; ONM), Carthusian (n = 1; IPN), Exotic shorthair (n = 1; IPN), PersianxRagdoll (n = 1; IPN), Siamese (n = 1; CTRL), Siberian (n = 1; IPN); Sphynx (n = 1; ONM), Tonkinese (n = 1; IPN). IPN (presumed) immune‐mediated polyneuropathy; ONM other neurological and neuromuscular disorder; CTRL non‐neurological control.

In the IPN‐group, 70.7% (29/41) of the cats were 2 years old or younger, 75.6% (31/41) of the cats were represented by Bengal (12/31), Domestic Shorthair (11/31) and British Shorthair (8/31) breeds, and male cats (75.6%; 31/41) were significantly overrepresented (*P* = .0220). Infectious disease serology revealed 9/41 IPN‐cats to be seropositive for FCoV (Persian (n = 2), British Shorthair (n = 2), Bengal, Carthusian, Domestic Shorthair, Exotic Shorthair and Maine Coon each n = 1), 2/40 to be antigen‐positive for FeLV (insufficient sample available for one cat), and none (0/41) to be seropositive for *Toxoplasma gondii* (IgM) or FIV.

Disease onset (recorded in 40/41 IPN‐cats) was in autumn (September to November) for 16/40 (40.0%) cats, in winter (December to February) for 10/40 (25.0%) cats, and in spring or summer for 7/40 (17.5%) cats each. Most samples (23/32 for which this information was available; 71.9%) were collected within 3 weeks of onset of clinical signs (range: 1‐100 days; median: 10 days).

The most common clinical presentation (Table 2) at time of examination, which in 18 of the 32 known cases coincided with the time‐point of serum acquisition, was ambulatory tetraparesis (17/41; 41.5%), followed by ambulatory paraparesis (13/41; 31.7%) and non‐ambulatory tetraparesis (24.4%). Hyporeflexia was reported in 35/40 (87.5%) cats for which this test result was recorded. Cranial nerve involvement was reported in 12/40 (30.0%) cats for which this information was available, typically being facial nerve paresis/paralysis (10/12) and with the affected nerve not specified in the remaining two cats. Six of 36 cats (16.7%) for which this information was available, presented with hyperaesthesia. One cat presented with respiratory compromise, and none with aspiration pneumonia.

**TABLE 2:** Clinical features of cats with immune‐mediated polyneuropathies (n = 41)

	Number	Present (%)
Gait	41	
Ambulatory paraparesis		13 (31.7)
Ambulatory tetraparesis		17 (41.5)
Non‐ambulatory paraparesis		1 (2.4)
Non‐ambulatory tetraparesis		10 (24.4)
Spinal reflexes		
Hyporeflexia	40	35 (87.5)
Areflexia	39	6 (15.4)
Hyperaesthesia	36	6 (16.7)
Involvement of cranial nerves	40	12 (30.0)
Respiratory compromise	38	1 (2.6)
Electrophysiological investigations		
Fibrillation potentials	33	29 (87.9)
Decreased MNCV	23	18 (78.2)
Dispersed CMAPs	22	16 (72.7)
Decreased CMAP amplitudes	24	21 (87.5)
Conduction block	18	4 (22.2)

Abbreviations: CMAP, compound muscle action potential; MNCV, motor nerve conduction velocity.

In electrophysiological investigations, 29/33 (87.9%) of IPN‐cats showed abnormal spontaneous myofibre activity on EMG (fibrillation potentials), whilst 18/23 (78.3%) cats exhibited decreased motor nerve conduction velocity, 21/24 (87.5%) cats had decreased CMAP amplitudes, 16/22 (72.7%) had temporal dispersion of CMAPs and 4/18 (22.2%) cats had reported evidence of a conduction block.

With regards to preceding events, two (of the 38 cats where known; 5.3%) had recently been vaccinated, one cat (2.6%) exhibited concomitant respiratory signs and one cat (2.6%) exhibited concomitant gastrointestinal disease.

Clinical presentations/diagnoses for the ONM group were seizures (n = 5), exercise intolerance due to myasthenia gravis (n = 2), head sway (n = 1) and a tetanus‐like presentation (n = 1).

### 3.2 Anti‐glycolipid antibody serology

**FIGURE 1:**
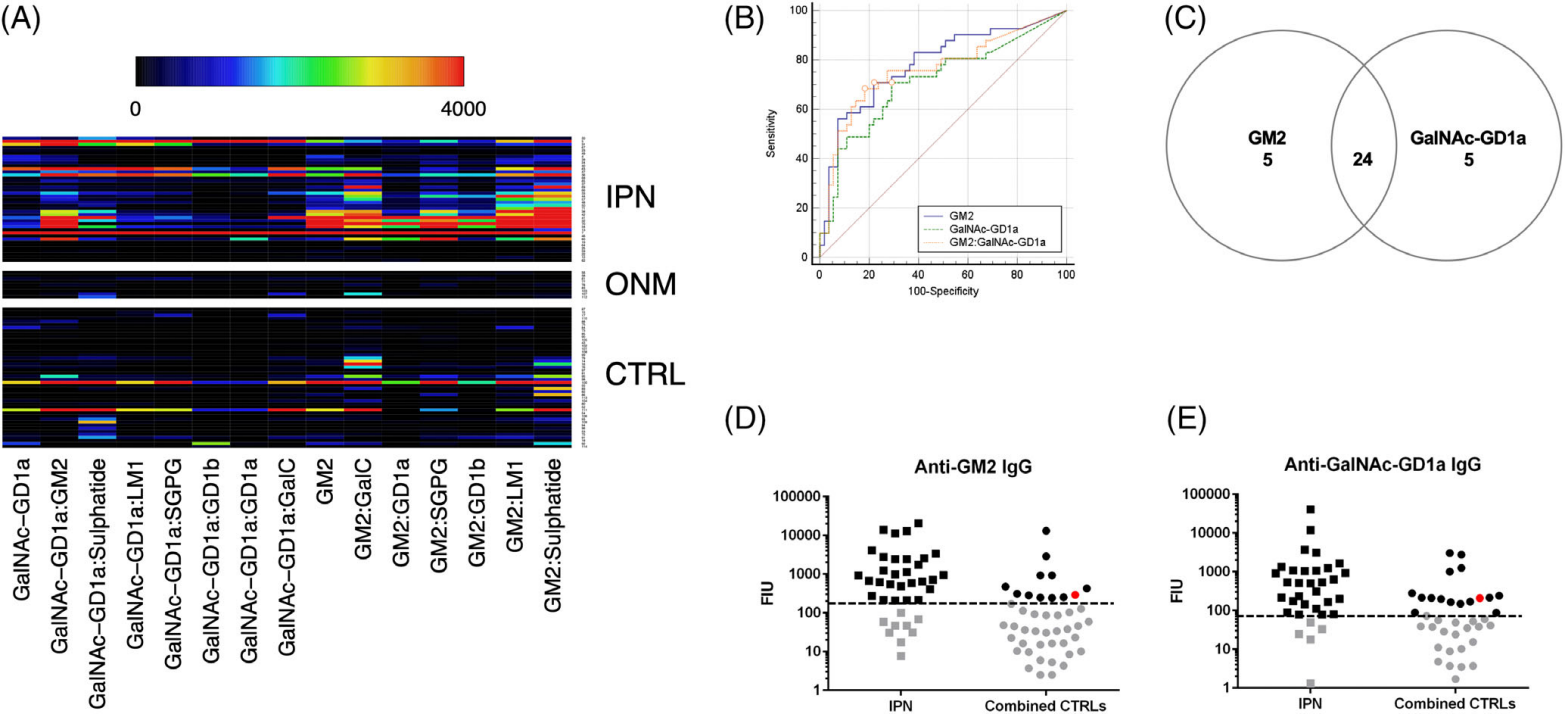
Serum IgG antibodies against glycolipids GalNAc‐GD1a and GM2 and their 1:1 heteromeric complexes in cats with immune‐mediated polyneuropathies (IPN; n = 41), other neurological and neuro‐muscular disorders (ONM; n = 9) and non‐neurological control cats (CTRL; n = 46). A, Heat map showing serum IgG anti‐glycolipid antibodies (AGAbs) binding to GalNAc‐GD1a and GM2 and some of their 1:1 heteromeric complexes (n = 15) which can be used to discriminate between IPN and control groups in this serological assay. Each column represents a unique glycolipid target, whilst each row represents a cat serum. The fluorescence intensity unit (FIU) intensity increases from black (0) through the rainbow scale to red (≥4000). Large numbers of IPN‐cats exhibit these AGAbs, whilst only individual cats in the control groups also exhibit such AGAbs. B, Receiver operating characteristic (ROC) curves showing anti‐GM2 IgG AGAbs to have a higher combined sensitivity and specificity when compared to anti‐GalNAc‐GD1a AGAbs or a combination of these. C, Venn diagram displaying the total number of positive IPN samples (FIU > threshold) for GM2 and/or GalNAc‐GD1a. A large proportion of IPN‐cats (82.9%) exhibit anti‐GM2 and/or anti‐GalNAc‐GD1a AGAbs; the remaining sera (7/41; 17.1%) are below cut‐off threshold for both these AGAbs. D, Dotplot for serum IgG AGAbs against glycolipid GM2. The dashed line represents the threshold cut‐off value (170.0 FIU). Anti‐GM2 IgG AGAbs above threshold cut‐off value are present in 29/41 IPN‐cats and in 12/55 combined control cats. The red dot represents the single positive ONM‐cat and all values below the threshold are displayed as grey shapes. E, Dotplot for serum IgG AGAbs against glycolipid GalNAc‐GD1a. The dashed line represents the threshold cut‐off value (72.3 FIU). Anti‐GalNAc‐GD1a IgG AGAbs above threshold cut‐off value are present in 29/41 IPN‐cats and in 16/55 combined control cats. The red dot represents the single positive ONM‐cat and all values below the threshold are displayed as grey shapes.

Screening sera from IPN, ONM and CTRL groups for the presence of IgG antibodies against a panel of 10 individual glycolipids and their associated 1:1 heteromeric complexes on the array platform, showed IPN‐sera to frequently contain anti‐GM2 antibodies (29/41) and its heteromeric complexes (Figure 1A, C, D), and overall lacking any specific grouping of AGAbs (Supplementary Figure S1). In most of the anti‐GM2 IgG positive sera (24/29; 82.8%), IgG AGAbs were also detected against GalNAc‐GD1a, albeit, frequently at lower FIU values (Figure 1C, E). Pairwise comparison of the GM2 and GalNAc‐GD1a ROC curve area under the curve (AUC; 0.784 vs 0.712) demonstrated anti‐GM2 IgG AGAbs as the superior target for the identification of IPN‐cats when compared to the combined controls (*P* = .0493; Figure 1B). When identifying the top 10 IgG targets by ROC analysis (giving equal weight to both sensitivity and specificity), all antibody targets contained one or both of these glycolipids, with combined sensitivity and specificity ranging from 154.3‐137.9 (Table 3). Pairwise comparison of ROC curves of the top five targets revealed no significant difference of the AUC of these targets (ranging from 0.732‐0.784; *P* > .05).

**TABLE 3:** Top 10 IgG serum anti‐glycolipid antibody targets for discriminating cats with immune‐mediated polyneuropathies from cats with other neurological and neuromuscular disorders and neurologically normal cats

Target	FIU cut‐off	Sensitivity (95% CI)	Specificity (95% CI)	Sensitivity + Specificity
GM2: LM1	>371.7	63.4 (46.9‐77.9)	90.9 (80.0‐97.0)	154.3
GM2:GD1a	>89.2	61 (44.5‐75.8)	92.7 (82.4‐98.0)	153.7
GM2:GalNAc‐GD1a	>130	68.3 (51.9‐81.9)	81.8 (69.1‐90.9)	150.1
GM2	>170	70.7 (54.5‐83.9)	78.2 (65.0‐88.2)	148.9
GM2:SGPG	>186.5	63.4 (46.9–77.9)	85.5 (73.3‐93.5)	148.9
GM2:Sulphatide	>908.5	61 (44.5–75.8)	85.5 (73.3‐93.5)	146.5
GM2:GD1b	>95.7	56.1 (39.7‐71.5)	89.1 (77.8‐95.9)	145.2
GalNAc‐GD1a	>72.3	70.7 (54.5‐83.9)	70.9 (57.1‐82.4)	141.6
GM2:GalC	>174.3	70.7 (54.5‐83.9)	67.3 (53.3‐79.3)	138
GalNAc‐GD1a:GD1a	>8.7	56.1 (39.7‐71.5)	81.8 (69.1‐90.9)	137.9

*Note*: CI, confidence interval, +LR Likelihood ratio (= sensitivity/(1 − specificity)), −LR Likelihood ratio (= (1 − specificity)/specificity).

**FIGURE 2:**
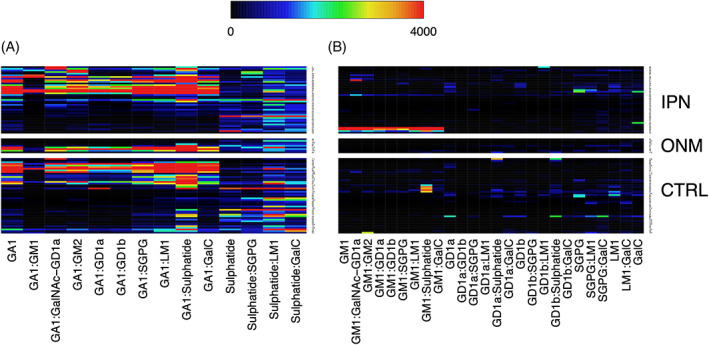
Serum IgG antibodies against glycolipids GA1, sulphatide, GM1, GD1, GD1b, SGPG, LM1 and galactocerebroside and some of their 1:1 heteromeric complexes in cats with immune‐mediated polyneuropathies (IPN; n = 41), other neurological and neuro‐muscular disorders (ONM; n = 9) and non‐neurological control cats (CTRL; n = 46). A, Heat map showing serum IgG antibodies against glycolipids GA1 and sulphatide and some of their 1:1 heteromeric complexes in cats. Each column represents a unique glycolipid target, whilst each row represents a cat serum. The fluorescence intensity unit (FIU) intensity increases from black (0) through the rainbow scale to red (≥ 4000). IgG anti‐glycolipid antibodies AGAbs are present at comparable levels across all groups and are therefore non‐discriminating biomarkers in this cohort of cats. B, Heat map showing serum IgG antibodies against glycolipids GM1, GD1a, GD1b, SGPG, LM1 and galactocerebroside and some of their 1:1 heteromeric complexes in cats. IgG AGAbs are either absent or infrequently present at comparable levels across all groups and are therefore non‐discriminating biomarkers in this cohort of cats.

Other AGAbs such as anti‐GA1 and/or ‐sulphatide IgG antibodies were present in a high proportion of IPN samples. However, these were deemed to be non‐specific for IPN‐cats, due to their ubiquitous presence across all sample groups (Figure 2A). Interestingly, this antibody pattern contrasts with the absence or low frequency of IgG AGAbs detected against glycolipids GM1, GD1a, GD1b, SGPG, LM1 and GalC (Figure 2B), other than when antibodies against such targets formed a part of heteromeric complexes with glycolipids GM2 or GalNAc‐GD1a (see above).

### 3.3 Correlation of serology with demographical, epidemiological and clinical parameters

Neither the patient demographics, nor the epidemiological factors, clinical features (ie, ambulatory status, hyporeflexia, areflexia, hyperaesthesia, involvement of cranial nerves or respiratory compromise), sampling intervals, the presence of fibrillation potentials or the infectious disease status represented a positive predictive factor for the presence of anti‐GM2 or anti‐GalNAc‐GD1a AGAbs.

A comprehensive analysis (lacking correction for multiple testing due to the relatively small dataset) and assessing all heteromer‐AGAbs, identified only hyperaesthesia and young age (≤2 years) as potential predictive factors for a number of anti‐GM2 heteromers (LM1 and SGPG) and anti‐GalNAc‐GD1a heteromers (galactocerebroside), respectively.

Finally, the proportion of IPN‐cats exhibiting anti‐GM2:GalC and anti‐GM2:GalNAc‐GD1a AGAbs was significantly greater in Bengals (*P* = .0082) and Bengals combined with British Shorthair cats (*P* = 0.0431), respectively, when compared to other breeds.

### 3.4 Anti‐GM2 and anti‐GalNAc‐GD1a AGAbs in control cats

One of the nine ONM‐cats (11.1%) and 11/46 CTRL‐cats (23.9%) exhibited anti‐GM2 serum IgG AGAbs, and 1/9 ONM‐cats (11.1%) and 15/46 CTRL‐cats (32.6%) exhibited anti‐GalNAc‐GD1a antibodies.

The clinical diagnosis for the single ONM‐cat exhibiting both anti‐GM2 and anti‐GalNAc‐GD1a IgG AGAbs was myasthenia gravis. This diagnosis was reached following electrophysiological investigations, but the anti‐nicotinic acetylcholine receptor antibody titre was within normal limits. A positive clinical response to corticosteroid therapy was reported in this case.

## 4 DISCUSSION

A recent large international multicentre study described the demographics and clinical features of cats clinically and histologically diagnosed with presumed immune‐mediated polyneuropathies.^16^ The cohort examined in the current study had similar clinical features, with over‐representation of young cats (typically ≤2 years old), male sex, and specific breeds comprising Bengal, Domestic and British Shorthair cats. We thus consider our cohort typically representative of the IPN diagnostic grouping.

Screening of the entire IPN‐cohort for underlying infectious disease, which represents a major differential aetiology for peripheral nerve disease in young cats, did not provide any evidence of infection with FIV or *Toxoplasma gondii*. Experimentally, an infection with FIV has been shown to be associated with a peripheral neuropathy.^20^ Toxoplasmosis, which in cats more typically causes encephalitis and myelitis,^21^ has been reported in an individual case with (distal) peripheral neuropathy.^22^ Only 2/40 tested IPN‐cats were positive for an infection with FeLV, which may in a chronic scenario cause myelopathy in cats^23^ and may also exhibit peripheral nervous system involvement following the development of virus‐induced lymphoma.^24^ The most recent (2016) Europe‐wide serological FeLV‐prevalence reported for cats taken to veterinary facilities is 2.3%, with higher rates observed in Italy (5.7%),^25^ where both the positive cats originated from in this study. Considering the requirement of chronicity for development of disease, the FeLV‐infection was considered incidental for the 7‐month‐old IPN‐cat. However, despite a lack of any other supporting signs or test results, a contributing factor to the development of a polyneuropathy could not be categorically ruled out for the 7‐year‐old cat that was FeLV positive. Finally, 9/41 IPN‐cats were seropositive for FCoV and these cats were mainly represented by purebred cats (8/9). The seroprevalence for FCoV‐specific antibodies varies substantially between catteries (up to 90%) and single cat households (up to 50%). The fact that purebred cats appear more susceptible to FCoV‐infection in general, that systemic infection with the (mutated) FCoV resulting in feline infectious peritonitis (opposed to purely gastrointestinal disease associated with the non‐mutated form of FCoV) only rarely affects the peripheral nervous system,^24^, ^26^ and that all of the seropositive cats in this study lacked any other supportive signs of feline infectious peritonitis, makes the seropositivity likely to be coincidental and unrelated to the diagnosis of IPN.

Reports of preceding or coinciding events, such as vaccinations, gastrointestinal or respiratory disease were sparse, thereby not supporting a clinically evident association with development of disease. However, more focused research with larger case numbers would be required to investigate whether a specific association may exist for these cases.

Interestingly, nearly two‐thirds of the IPN‐cats had a disease onset in the autumn (25%) and winter (40%) months, similar to observed in previous studies on immune‐mediated peripheral neuropathies (ACP) in dogs.^14^, ^27^

The IPN‐cats in this study possessed serum anti‐GM2 and anti‐GalNAc‐GD1a antibodies as the most distinct AGAb marker, highly similar to our previous findings in ACP‐dogs.^14^ Antibodies to both these targets reached a higher sensitivity, yet slightly lower specificity, when compared to the ACP‐dogs. Also as seen in ACP‐dogs, many IPN‐cats possessed both anti‐GM2 and anti‐GalNAc‐GD1a AGAbs, yet with lower binding levels for the latter. This is most likely due to cross‐reactivity between anti‐GM2 and anti‐GalNAc‐GD1a AGAbs, since these glycolipids contain an identical terminal trisaccharide moiety.^10^, ^28^

Co‐existing anti‐GM2 and anti‐GalNAc‐GD1a (albeit IgM) AGAbs have been reported in human patients suffering from chronic demyelinating neuropathy with sensory ataxia,^12^ from pure motor chronic demyelinating neuropathy,^10^ and from demyelinating neuropathy characterised by slow progression, frequent facial nerve involvement (occasionally also observed in this cohort of cats) and sensory deficits.^11^ Human patients with GBS may also possess anti‐GM2 and anti‐GalNAc‐GD1a IgG antibodies,^7^, ^8^, ^9^ but these more rarely co‐occur than is seen in ACP and IPN, suggesting potential differences in antibody fine specificity between cats/dogs and humans.

Electrophysiological investigations, conducted in 34/41 of the IPN‐cats in this cohort, revealed spontaneous myofibre activity on EMG, decreased motor nerve conduction velocities, dispersed and decreased compound muscle action potentials, and variable conduction blocks. These changes are consistent with previous reports in the literature and support the presence both of demyelination and axonal loss in IPN‐cats.^5^, ^6^, ^16^ Conversely, histological examinations conducted on peripheral nerve biopsies taken from IPN‐cats show evidence of demyelination and remyelination, less commonly axonal degeneration and regeneration, and variable inflammatory infiltrates. Muscle biopsies from affected cats commonly show a loss of myelinated intramuscular nerve fibres with or without mononuclear infiltrates.^4^, ^5^, ^6^, ^29^ These features provide a close pathophysiological resemblance between IPN and some human polyneuropathies in which anti‐GM2 and anti‐GalNAc‐GD1a antibodies are found.

The heterogenic clinical signs in cats with IPN could be explained by the various potential different binding sites for anti‐GM2 and anti‐GalNAc‐GD1a antibodies. Our previous investigations in dogs have confirmed the presence of ganglioside GM2 in peripheral nerve and localised it to abaxonal Schwann cell membranes, less so to axonal regions.^15^ Similarly, the presence of GM2 has previously been demonstrated in feline sciatic nerve,^28^ and with immunohistochemical investigations has been located to the abaxonal Schwann cell membranes (data not shown). Selective Schwann cell injury has been shown to be associated with myelin fragmentation either affecting parts or the entire internode,^30^ and secondary axonal degeneration is commonly observed in demyelinating neuropathies.^31^ Similarly, in human peripheral nerves and nerve roots, anti‐GM2 antibody‐containing sera also stains abaxonal regions of the myelin sheath/Schwann cells, and less so the axons,^32^ whilst anti‐GalNAc‐GD1a antibodies stain the axonal regions of myelin and the periaxonal regions at the nodes of Ranvier in ventral nerve roots, and intramuscular nerves.^33^ Furthermore, antibodies binding the shared terminal moiety of GM2 and GalNAc‐GD1a have been shown to bind both the presynaptic Schwann cells and nerve fibres of the murine neuromuscular junction.^34^

Selective targeting of not only two different components, but also two geographically quite different regions of the peripheral nervous system might not only explain the variations in clinical presentation between IPN‐cats, similar to recently reported in human patients with anti‐GM2 antibodies,^9^ but also recovery times for IPN‐cats, which in recent literature range from a few days to 17 months.^16^ At the same time, such a heterogeneity in targets and regions might provide a potential explanation for the relative lack of distinct AGAb‐binding patterns observed between breeds, ages and phenotypes, which lies in stark contrast to more typical GBS‐subtypes and their distinct AGAb‐profiles,^35^ and might further also explain the lack of correlation of certain IPN‐breeds to histological features.^29^

Whilst being unable to directly demonstrate causality, in combination with experimental work conducted on potential pathophysiological roles of AGAbs in GBS,^36^, ^37^ the results of this study strongly suggest that the anti‐GM2 and anti‐GalNAc‐GD1a antibodies we have observed in IPN‐cats are likely to be pathogenic factors, as well as suitable biomarkers. Feline IgG1 is capable of fixing complement and thus acting mechanistically in IPN to drive complement‐mediated nerve injury.^38^ Owing to the absence of serial samples, we were not able to assess serial titres over time and ethical considerations further preclude pathophysiological analysis of causality in modelling studies directly carried out in cats.

Finally, a number of cats in the non‐neurological control population exhibited anti‐GM2 and/or anti‐GalNAc‐GD1a antibodies above the cut‐off threshold. Albeit slightly higher in frequency, this observation corresponds to our and other previous observations in neurologically normal dogs and humans, in which AGAbs are also occasionally found.^14^, ^32^ Many AGAbs exist in the normal natural antibody repertoire, notably but not restricted to anti‐GA1 and ‐sulphatide antibodies as reported herein for cats. It is therefore imperative to carefully consider assay thresholds, choices of antigens and disease control groups when conducting studies on AGAbs.

The one ONM‐cat that exhibited both anti‐GM2 and anti‐GalNAc‐GD1a AGAbs was diagnosed with myasthenia gravis, but it remains possible that this case may have been affected by an alternative or concurrent IPN, particularly given the normal acetylcholine receptor antibody titre.

## 5 CONCLUSION

This study indicates that in cats clinically diagnosed with immune‐mediated peripheral neuropathies, anti‐GM2 and anti‐GalNAc‐GD1a IgG antibodies, may potentially serve as useful biomarkers for disease, with anti‐GM2 AGAbs being considered the most favourable and simple antibody test to conduct.

Considering the histological and electrophysiological findings reported for cats with immune‐mediated peripheral neuropathies, it seems likely that, as is the case in dogs and humans, anti‐GM2 and anti‐GalNAc‐GD1a AGAbs are a contributing cause of AGAb‐mediated demyelinating neuropathy on which further pathophysiological studies are warranted.

## CONFLICT OF INTEREST

No conflicts of interest have been declared.

## Supporting information


**Figure S1.** Summary of anti‐glycolipid antibody (AGAb) levels measured in cats with immune‐mediated polyneuropathies (IPN). Antibody levels in fluorescence intensity units (FIU) were log transformed before calculation of Z‐score across the population of IPN‐cats. Antibodies (listed on ordinate) and cats (listed on right abscissa) were grouped by complete Euclidean clustering. Overall, no distinct grouping of AGAb specificities in relation to the annotations (depicted on the left abscissa) breed, sex, age, cranial nerve (CN) involvement and gait status (ambulatory/non‐ambulatory paraparesis/tetraparesis) are observed.

**Video S1.** 
