# Supplementary figures and images for: Serum anti‐GM2 and anti‐GalNAc‐GD1a ganglioside IgG antibodies are biomarkers for immune‐mediated polyneuropathies in cats
Source: J Peripher Nerv Syst. 2023 Jan 16;28(1):32–40. doi: 10.1111/jns.12529 (PMC10946849; doi:10.1111/jns.12529)

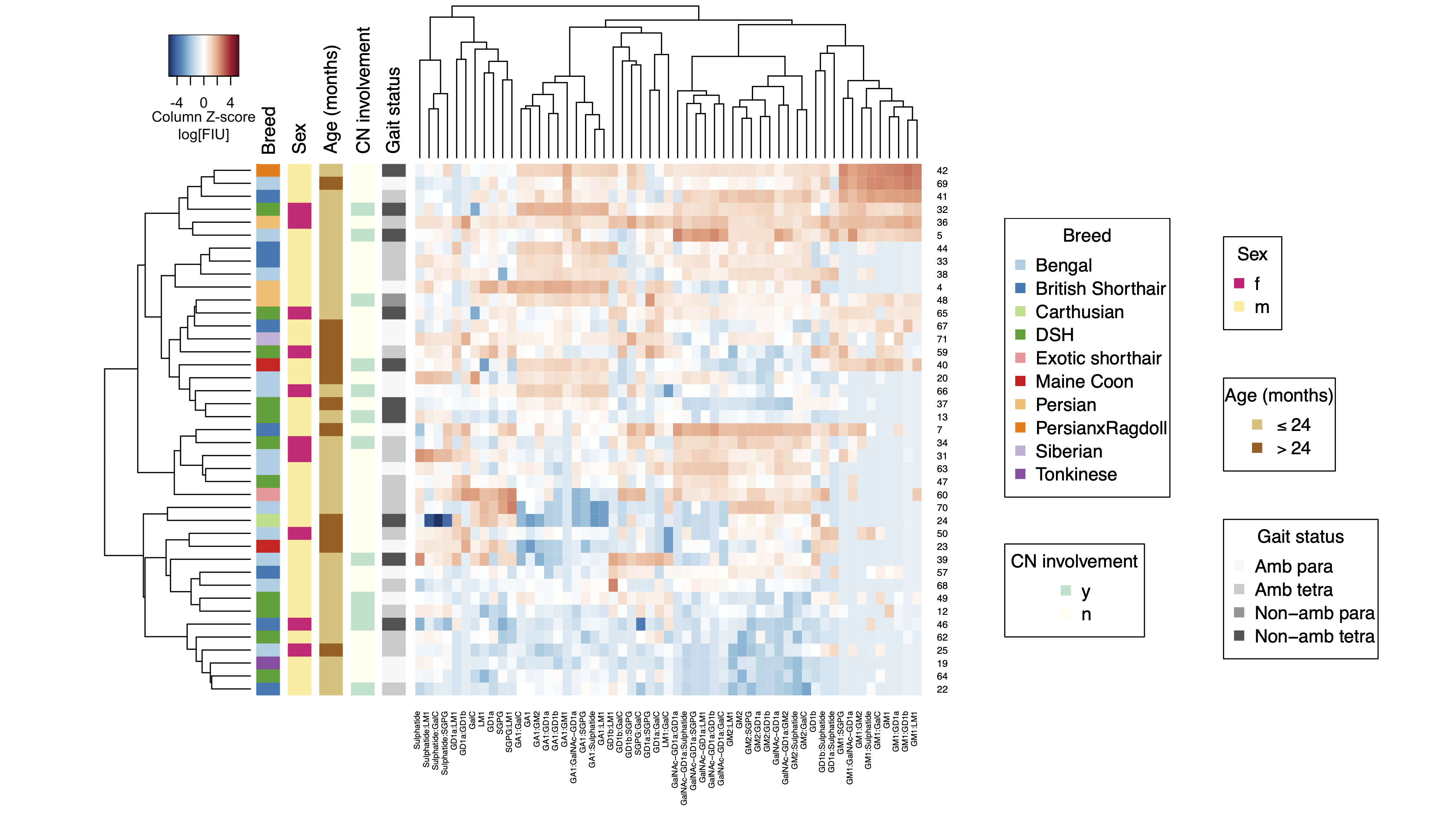

Supplement: Supplementary file 1 — Figure S1. Summary of anti‐glycolipid antibody (AGAb) levels measured in cats with immune‐mediated polyneuropathies (IPN). Antibody levels in fluorescence intensity units (FIU) were log transformed before calculation of Z‐score across the population of IPN‐cats. Antibodies (listed on ordinate) and cats (listed on right abscissa) were grouped by complete Euclidean clustering. Overall, no distinct grouping of AGAb specificities in relation to the annotations (depicted on the left abscissa) breed, sex, age, cranial nerve (CN) involvement and gait status (ambulatory/non‐ambulatory paraparesis/tetraparesis) are observed. [file JNS-28-32-s001.tiff]
